# Expression of Concern: LncRNA DQ786243 contributes to proliferation and metastasis of colorectal cancer both *in vitro* and *in vivo*

**DOI:** 10.1042/BSR-20160048_EOC

**Published:** 2021-04-06

**Keywords:** colorectal cancer (CRC), invasion, long non-coding ribonucleic acid (LncRA), migration

The Editorial Office has been made aware of potential issues surrounding the scientific validity of this paper, hence has issued an expression of concern to notify readers whilst the Editorial Office investigates.

